# The prevalence of clinically relevant delayed intracranial hemorrhage in head trauma patients treated with oral anticoagulants is very low: a retrospective cohort register study

**DOI:** 10.1186/s13049-024-01214-0

**Published:** 2024-05-10

**Authors:** Lars André, Anders Björkelund, Ulf Ekelund, Tomas Vedin, Jonas Björk, Jakob Lundager Forberg

**Affiliations:** 1 Department of Clinical Sciences, Lund University, Lund, Sweden; 2 Department of Emergency Medicine, Helsingborg Hospital, Helsingborg, Sweden; 3 Centre for Environmental and Climate Science, Lund University, Lund, Sweden; 4 Department of Internal medicine and Emergency care, Skåne University Hospital, Lund, Sweden; 5 Department of Clinical Sciences, Lund University, Malmö, Sweden; 6 Department of Surgery, Skåne University Hospital, Malmö, Sweden; 7 Department of Laboratory Medicine, Lund University, Lund, Sweden; 8 Skåne University Hospital, Forum South, Clinical Studies Sweden, Lund, Sweden

**Keywords:** Head trauma, Traumatic brain injury, Delayed intracranial hemorrhage, Oral anticoagulation

## Abstract

**Background:**

Current guidelines from Scandinavian Neuro Committee mandate a 24-hour observation for head trauma patients on anticoagulants, even with normal initial head CT scans, as a means not to miss delayed intracranial hemorrhages. This study aimed to assess the prevalence, and time to diagnosis, of clinically relevant delayed intracranial hemorrhage in head trauma patients treated with oral anticoagulants.

**Method:**

Utilizing comprehensive two-year data from Region Skåne’s emergency departments, which serve a population of 1.3 million inhabitants, this study focused on adult head trauma patients prescribed oral anticoagulants. We identified those with intracranial hemorrhage within 30 days, defining delayed intracranial hemorrhage as a bleeding not apparent on their initial CT head scan. These cases were further defined as clinically relevant if associated with mortality, any intensive care unit admission, or neurosurgery.

**Results:**

Out of the included 2,362 head injury cases (median age 84, 56% on a direct acting oral anticoagulant), five developed delayed intracranial hemorrhages. None of these five cases underwent neurosurgery nor were admitted to an intensive care unit. Only two cases (0.08%, 95% confidence interval [0.01–0.3%]) were classified as clinically relevant, involving subdural hematomas in patients aged 82 and 87 years, who both subsequently died. The diagnosis of these delayed intracranial hemorrhages was made at 4 and 7 days following initial presentation to the emergency department.

**Conclusion:**

In patients with head trauma, on oral anticoagulation, the incidence of clinically relevant delayed intracranial hemorrhage was found to be less than one in a thousand, with detection occurring four days or later after initial presentation. This challenges the effectiveness of the 24-hour observation period recommended by the Scandinavian Neurotrauma Committee guidelines, suggesting a need to reassess these guidelines to optimise care and resource allocation.

**Trial registration:**

This is a retrospective cohort study, does not include any intervention, and has therefore not been registered.

## Background

Head trauma is common and often leads to emergency department (ED) visits, ranging from mild concussions to severe, life-threatening injuries with intracranial hemorrhage (ICH), necessitating early detection and treatment. The initial symptoms of ICH may be subtle and resemble those of a concussion, which makes management in the ED challenging. Even when an initial computed tomography (CT) scan shows no ICH, patients may still develop a delayed ICH (d-ICH), specifically those on oral anticoagulants (OAC), presenting additional challenges. Management strategies to prevent overlooking d-ICH include repeat CT scanning, prolonged observation, and individual risk assessments.

In Sweden there are five commercially available OAC drugs, one being the vitamin K antagonist warfarin and the other four are direct oral anticoagulants (DOAC): dabigatran (direct thrombin inhibitor), rivaroxaban (factor Xa inhibitor), edoxaban (factor Xa inhibitor) and apixaban (factor Xa inhibitor). Oral drugs that inhibit thrombocyte aggregation, such as aspirin, non-steroidal anti-inflammatory drugs, and adenosine diphosphate receptor inhibitors, are not included in the term OAC.

The use of OAC treatment, particularly DOACs, is on a steady rise in recent years, especially among the elderly [1–3]. At the same time, studies have shown an increasing incidence of head injuries among those older than 65 years [4]. A third of these patients experience a fall at least once per year, with 10% of these falls resulting in a head injury [5].

Hence, head injuries in patients with OAC therapy are common and expected to increase in the coming years, placing a greater strain on our emergency departments and hospitals. Consequently, the development and updating of evidence-based clinical guidelines becomes crucial to effectively manage these patients with increased risks of traumatic ICH and d-ICH.

Most current guidelines for head injuries – including the New Orleans Criteria [6], Canadian CT Head Rule [7], EAST’s practice management guidelines [8], French guidelines for mild Traumatic Brain Injury [9], the Austrian interdisciplinary consensus statement [10] and the Scandinavian Neurotrauma Committee Guidelines (SNC) [11], mandate a head CT scan for patients on anticoagulation. Recently updated NICE guidelines [12] state that a head CT is to be considered, but not mandatory, for patients treated with OAC. In addition to an initial head CT, the Austrian consensus document and SNC guidelines both advise a 24-hour observation period for these patients, even if their initial CT scan is interpreted as normal, as a means not to miss cases of d-ICH [10, 11].

The recommendation to observe all head trauma patients on OAC treatment requires significant ED and in-hospital resources and may lead to adverse outcomes, particularly among elderly patients [13–16]. In addition, most d-ICH cases do not necessitate management changes [17], and it may be posited that only clinically relevant d-ICH, requiring neurosurgical intervention, intensive care unit (ICU) admission, or leading to death, warrant detection. The aim of this study was to analyze the incidence, and time to diagnosis, of clinically relevant d-ICH in a Scandinavian ED setting.

## Method

A retrospective cohort study was conducted in Region Skåne, Sweden, where the hospital system serves a population of 1.3 million inhabitants. This encompasses eight emergency departments: three general EDs, three community EDs, and two academic center EDs, one of which is a neurosurgical center.

Data on adult ED visits during 2017 and 2018 were collected from local, regional, and national healthcare registers, including information from the hospital system’s medical records and national data from the National Board of Health and Welfare and the National Pharmacy Register.

Data was retrieved for adult patients, aged 18 years or older, presenting to an ED with head trauma registered as their chief complaint by triage staff in the ED information system. Patients were included in the study as an OAC treated patient if they had redeemed a prescription of any OAC at a Swedish pharmacy within 90 days prior to their ED visit.

Radiology reports for all CT scans capable of detecting ICH within 30 days of initial ED presentation were retrieved. This included standard non-contrast head/brain CT as well as CTs focused on neurocranium, facial bones, orbits, and cerebral angiography. Reports were categorised binarily as positive or negative for ICH after both manual and automatic review. To avoid ambiguity between d-ICH and delayed diagnostics we excluded ED visits where there was no CT performed or if the first CT scan was performed after 24 h of ED arrival.

Neurosurgical interventions, recorded nationally per the Swedish system “Classification of Healthcare Interventions (KVÅ)”, were identified using a compiled list of ICH-related intervention codes (see *Appendix*, Table 1). Registration of any of these codes indicated that the patient had undergone neurosurgery and included all types of craniectomy, duraplasty, intracranial pressure monitoring and hematoma evacuation as well as intracranial vascular and ventricular surgery.

Delayed ICH was defined as the finding of ICH within 30 days of head trauma, which was not identified on the first CT scan performed within 24 h of ED arrival, and without known repeat head trauma occurring since the preceding CT scan.

Hospital records and ambulance documentation were reviewed for suspected d-ICH cases. Cases were classified as not being d-ICH if there was documentation of repeat head trauma since the initial CT scan. Admission to an intensive care unit was verified through local regional hospital records.

Nationally registered mortality data was retrieved using national identity numbers. This data included cause of death and was used to identify whether patients had died due to traumatic brain injury outside of Region Skåne hospital system, where we would be unable to access hospital records.

The primary outcome, classified as clinically relevant d-ICH, was defined as d-ICH associated with neurosurgery, death, or any ICU treatment within 30 days of the index ED visit.

Given the study’s basis on ED visits, individual patients could be included multiple times if subsequent ED visits met inclusion criteria.

### Statistics

Patient characteristics were reported using descriptive statistics. Data was assessed for normal distribution using visual assessment of histograms. Nonnormally distributed data were reported as medians with 25–75%, exclusive, quartiles range (QR). Data was analysed using Microsoft Excel 365. The 95% confidence interval (CI) for proportions was calculated with the Clopper-Pearson exact method using the Epitools epidemiological calculator [18].

## Results

**Table 1:** Patients characteristics and outcomes

		All *n* = 2,362	Warfarin use *n* = 1,017^‡^	DOAC use *n* = 1,310^‡^
**PATIENT PARAMETERS**	**Value type**			
Age years	median (QR)	84 (77–89)	85 (79–90)	83 (76–89)
Women	n (%)	1,290 (54.6%)	535 (52.6%)	738 (56.3%)
Residency in Region Skåne	n (%)	2,333 (98.8%)	1,004 (98.7%)	1,294 (98.8%)
Ambulance arrival	n (%)	1,713 (72.5%)	737 (72.4%)	946 (72.2%)
INR^†^	median (QR)	1.9 (1.2–2.6)	2.5 (2.1-3.0)	1.1 (1.0-1.3)
INR <2	n (%)	756 (32.0%)	115 (11.3%)	626 (47.8%)
INR ≥2 and ≤ 3	n (%)	541 (22.9%)	530 (52.1%)	11 (0.8%)
INR >3	n (%)	194 (8.2%)	192 (18.9%)	2 (0.2%)
**Anticoagulation** ^‡^				
Warfarin	n (%)	1,036 (43.9%)	1,017 (100%)	-
Dabigatran	n (%)	217 (9.2%)	-	211 (16.1%)
Rivaroxaban	n (%)	413 (17.5%)	-	394 (30.1%)
Apixaban	n (%)	725 (30.7%)	-	700 (53.4%)
Edoxaban	n (%)	7 (0.3%)	-	5 (0.4%)
**30-DAY OUTCOMES**				
All-cause mortality	n (%, [CI])	112 (4.7%, [3.9–5.7%])	49 (4.8%, [3.6–6.3%])	62 (4.7%, [3.6-6.0%])
Neurosurgery	n (%, [CI])	6 (0.3%, [0.1–0.6%])	5 (0.5%, [0.2–1.1%])	1 (0.1%, [0.0-0.4%])
d-ICH all	n (%, [CI])	5 (0.2%, [0.1–0.5%])	2 (0.2%, [0.0-0.7%])	3 (0.2%, [0.0-0.7%])
d-ICH with Mortality	n (%, [CI])	2 (0.08%, [0.01–0.3%]	0 (0.0%, [0.0-0.4%])	2 (0.15%, [0.02–0.6%])
d-ICH with ICU admission	n (%, [CI])	0 (0.0% [0.0-0.2%])	0 (0.0%, [0.0-0.4%])	0 (0.0%, [0.0-0.03%])
d-ICH with Neurosurgery	n (%, [CI])	0 (0.0% [0.0-0.2%])	0 (0.0%, [0.0-0.4%])	0 (0.0%, [0.0-0.03%])

**CI** : 95% confidence interval limits, Clopper-Pearson exact method

**QR** : Quartiles range, exclusive, 25–75%

**INR** : Prothrombin time, International Normalized Ratio

**DOAC**: Direct Oral Anticoagulant

**ICU**: Intensive Care Unit

^‡^35 cases had more than one redeemed oral anticoagulant prescription, likely representing a switch in therapy. These were omitted for separate statistics of Warfarin and DOAC groups. Neither of these patients had d-ICH findings.

^†^INR results were missing in 871 (36,9%) of the total cases; this includes 180 (17.7%) in the Warfarin group and 671 (51.2%) in the DOAC group

**Fig. 1:**
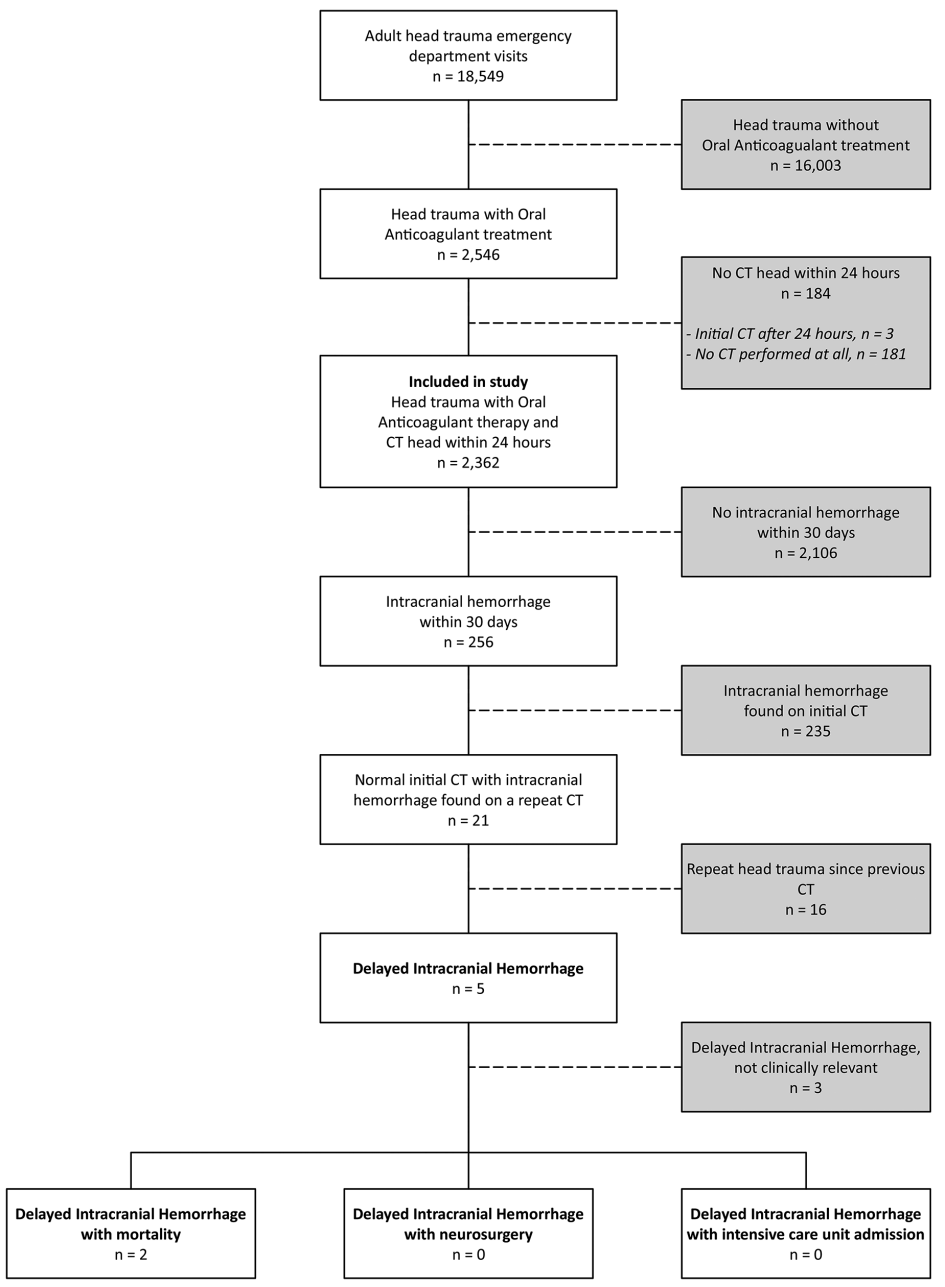
Flowchart showing inclusion and exclusion process of the entire study, and main outcomes, with 2,362 included emegency department visits due to head trauma and concomitant oral anticoagulation. Two patients were found to have clinically relevant delayed intracranial hemorrhage

Data from 630,275 ED visits were retrieved, with 18,549 being visits due to a chief complaint of head trauma. Of these, a total of 2,362 head trauma ED visits were included in the study. See Fig. 1 for the inclusion process and main results, and Table 1 for patient demographics and observed outcomes. The median age was 84 (QR 77–89), and the majority of the patients were treated with DOAC (56.1%), with Apixaban being the most common DOAC (30.7% of total).

Within 30 days of ED presentation, 256 cases of ICH were diagnosed. Of these, 21 initially had a negative CT scan at their respective index ED visit, but a repeated CT within 30 days detected ICH. Subsequent review of hospital and ambulance records for these 21 patients revealed that 16 had suffered a repeat head trauma after the initial ED visit. Consequently, only five cases were classified as d-ICH (0.2%, 95% CI [0.07–0.5%]) by our definition. Of the five patients with d-ICH, four presented with subdural hematomas (SDH), and one had a minor intracerebral hemorrhage. None of the d-ICH cases were identified within the initial 24-hour period. Instead, repeated CT scans identified d-ICH on day 4, 8, 11, 21 and 28 post index ED visit, respectively. Detailed case information is provided in Table 2. Review of hospital notes for the five patients with d-ICH revealed no ICU admissions.

During the study period, 325 patients had more than one ED visit due to head trauma: 232 had two visits, 59 had three visits, 23 had four visits, and 11 had five or more visits. None of the d-ICH cases had more than one ED visit registered as head trauma.

Through national registry data, we found that six patients underwent a neurosurgical intervention within 30 days, all with evacuation of SDHs, and all had findings of ICH on their initial CT scan. None of the five d-ICH cases underwent a neurosurgical procedure.

We observed an all-cause 30-day mortality of 4.7% (95% CI [3.9–5.7%]). Two patients with d-ICH died within 30 days of their initial ED visit, at 7 and 14 days after the index ED visit, respectively. In our dataset, 1.2% of patients were inhabitants in a geographic region not covered by the Region Skåne hospital system, and three of those died within 30 days, all with ICH on their initial CT scan. Cross-referencing national mortality data with local hospital records revealed no further mortality cases with any type of ICH as a listed cause of death.

In summary, we identified two cases (0.08%, 95% CI [0.01–0.3%]) of clinically relevant d-ICH due to associated mortality, but none underwent neurosurgery nor admitted to ICU.

**Table 2:** Clinical summaries of cases with delayed intracranial hemorrhage

Case number	Case description		ICH type and days to diagnosis*	Clinically relevant d-ICH
**1**	Anticoagulation	Rivaroxaban	SDH: acute with midline shift 4 days	**Yes** Death 7 days after head trauma
	Age	82 years		
	Notable case characteristics	Ground level fall. Severe dementia. Nursing home resident.		
	Hospital admission	No, discharged back to nursing home		
**2**	Anticoagulation	Rivaroxaban	SDH: chronic bilateral 7 days	**Yes** Death 14 days after head trauma
	Age	85 years		
	Notable case characteristics	Ground level fall. Dementia. Nursing home resident. Lower extremity fracture, rib fractures. Post-operative infection as cause of death.		
	Hospital admission	Yes, with surgery of fracture		
**3**	Anticoagulation	Warfarin	SDH: chronic bilateral 21 days	**No**
	Age	87 years		
	Notable case characteristics	Ground level fall. Upper extremity fracture.		
	Hospital admission	Yes		
**4**	Anticoagulation	Apixaban	SDH: chronic unilateral 28 days	**No**
	Age	79 years		
	Notable case characteristics	Fall down set of stairs. Skull and facial bone fractures.		
	Hospital admission	Yes		
**5**	Anticoagulation	Warfarin	Basal ganglia ICH 11 days	**No**
	Age	85 years		
	Notable case characteristics	Ground level fall. Arrhythmia.		
	Hospital admission	Yes, with cardiology evaluation		

**ICH**: Intracranial Hemorrhage

**d-ICH**: Delayed Intracranial Hemorrhage

**SDH**: Subdural hematoma

* Days from index emergency department visit to diagnosis of ICH

## Discussion

In our study of 2,362 ED visits involving head trauma and OAC therapy, we identified five cases of d-ICH, accounting for 0.2% of the visits (95% CI [0.07–0.5%]). Of these, two cases (0.08%, [95% CI 0.01–0.3%]) were classified as clinically relevant, due to their association with mortality, yet neither required ICU admission or neurosurgery. None of the observed d-ICH cases were diagnosed within the 24-hour observation period recommended by SNC and the Austrian consensus document. On the contrary, the few cases of d-ICH in our study were diagnosed four or more days after their initial ED visit, aligning with Chenoweth et al.’s findings where two of three d-ICH cases were detected after 24 h [19]. This suggests that the 24-hour observation period may be inadequate in detecting d-ICH. Our findings challenge the utility of currently recommended 24-hour observation period by the Scandinavian Neurotrauma Committee (SNC) guideline and the Austrian expert consensus document. Our results lend support to other guidelines that do not mandate such observation [6–9, 12].

Mandatory observation for d-ICH in the ED and in-hospital units carries significant costs and adds a burden on already strained healthcare resources. In 2012, the cost in the USA was estimated at 1,000,000 USD to detect a single case of d-ICH, and this cost is likely much higher now [20]. Additionally, the typical patient demographic for OAC therapy – older and frail individuals – may be harmed by even short-term hospitalizations [13–16]. Furthermore, other concerns could be present that are of higher individual value than in-hospital observation for the rare event of d-ICH.

Previous studies assessing the risk of d-ICH in anticoagulated patients have utilized diverse methodologies and patient populations, making it difficult to determine its true prevalence. The latest meta-analysis by Puzio et al., conducted after the introduction of direct oral anticoagulant (DOAC) therapies, reported a 2.3% incidence of d-ICH in patients treated with OAC who suffered a blunt head injury [21]. However, several more recent studies, reflecting the increased use of DOACs compared to warfarin, generally suggest incidence rates of ≤ 1% [22–26]. The variance in incidence rates between studies could be attributed not only to differences in follow-up methodology but also to misdiagnosis. For instance, Verschoof et al. found that 66% of their d-ICH cases were actually erroneous, with subtle ICH noted retrospectively on initial CT scans [22]. The advent of advanced CT technology may reduce the incidence of d-ICH by enabling the detection of minimal ICH on initial scans [27].

Our study, which observed a d-ICH incidence of 0.2% (95% CI [0.07–0.5%]), was not designed to find all cases of d-ICH, being a retrospective cohort without structured follow-up or imaging. However, our findings are in line with recent studies that report a low incidence of d-ICH.

Many cases of d-ICH are minor and often managed conservatively. Clinically important d-ICH, which requires neurosurgery or causes death, appear to be very rare. Puzio et al.’s meta-analysis indicated a crude risk of death from d-ICH at 0.33% and d-ICH-related neurosurgical interventions at 0.13% [21]. Several newer studies have shown that important interventions, such as neurosurgery, is extremely rare, with many reporting no neurosurgical interventions across various patient populations [14, 16, 23–26]. This aligns with the results of our study, where we found no d-ICH related neurosurgical interventions (0.0%, 95% CI [0.0-0.03%]) or ICU admissions (0.0%, 95% CI [0.0-0.03%]), and a low mortality rate (0.08%, [95% CI 0.01–0.3%]).

Given the low incidence of clinically relevant d-ICH, and considering that very few cases seem appropriate for neurosurgical intervention, it has been proposed that mandatory observation for OAC treated patients should be omitted [28]. This perspective is mirrored in more recently reviewed guidelines, like those from NICE and French recommendations, which do not advocate for routine observation following a negative initial CT [9, 12]. This opinion, reinforced by recent evidence including findings from this study, supports reconsidering the necessity of mandatory observation and calls for a revision of the SNC guidelines.

## Strengths and limitations

The study’s strengths include being the largest study, to our knowledge, that investigates clinically relevant outcomes in d-ICH among OAC-treated head trauma patients. It included a diverse and extensive cohort from two academic, three general, and three community EDs, which enhances the applicability of the results. Additionally, the use of Swedish nationwide registers ensures reliable follow-up data.

The study does have limitations. A primary concern is the reliance on prescription redemption to determine OAC use, which might not always reflect the actual ongoing treatment at the time of injury. This approach could include patients who had ceased OAC use for various reasons, such as compliance issues or the completion of a prescribed treatment course. This factor might lead to an underestimation of the actual rate of clinically relevant d-ICH. However, it is unlikely that the inclusion of such cases, which are probably few, significantly alters the overall interpretation of the study’s results.

Our study focused on patients with ‘head injury’ as the chief complaint registered by triage staff. This will lead to missing patients with head injuries registered under another major complaint, such as a hip fracture, syncope, or multi trauma, resulting in their non-inclusion in study. However, we believe this does not significantly impact our conclusions. Patients with other major issues, e.g. a hip fracture, would require in-hospital treatment and observation anyway.

This study focused on use of OAC treatment and did not evaluate use of thrombocyte inhibitors, neither on its own nor in combination with OAC. Findings should not be applied for patients treated with thrombocyte inhibitors.

We did not evaluate the mechanism of injury leading to head trauma. Investigating trauma energy as a predictor for clinically relevant d-ICH could potentially be valuable for further individual risk assessment. However, the rarity of d-ICH necessitates substantially larger studies to achieve adequate statistical power. In our data, d-ICH was identified only in cases of ground level falls, which could indicate that low-energy trauma increases the risk of developing d-ICH.

A minor limitation of our study is that 1.2% of the patients were not residents of the geographical area served by our hospital system, which could potentially affect the completeness of our follow-up data. There is also a possibility that some patients may have been diagnosed with d-ICH in other regions where we could not access their medical records. However, since data on neurosurgical interventions and mortality were captured in nationwide registries, our analysis would not have missed any cases related to these outcomes. The only potentially missed cases would be those of d-ICH that required admission for ICU care.

The majority of patients in our study did not undergo repeat CT scan, as this is not routinely performed in line with SNC guidelines. Consequently, this study should not be used to interpret the true incidence of d-ICH in general but only of those cases causing clinical concern. The prevalence of clinically relevant d-ICH, which is the primary objective of our study, should remain unaffected by this limitation. For the same reason, the high ratio of d-ICH being clinically relevant (two out of five, 40%) should be interpreted with caution, as the denominator is likely larger.

## Conclusion

In patients with head trauma, on oral anticoagulation, the incidence of clinically relevant delayed intracranial hemorrhage was found to be less than one in a thousand, with detection occurring four days or later after initial presentation. This challenges the effectiveness of the 24-hour observation period recommended by the Scandinavian Neurotrauma Committee guidelines, suggesting a need to reassess these guidelines to optimise care and resource allocation.

## Data Availability

Due to the nature of this retrospective cohort study, data sharing is restricted. The data contain sensitive patient information protected under national privacy regulations and patient confidentiality agreements. As such, the dataset for this study is not publicly available. Access is limited to the research team to maintain patient privacy and adhere to ethical standards. Researchers interested in the study methodology or other non-sensitive materials can contact the corresponding author.

## Appendix

**Appendix Table 1:** Neurosurgical interventions according to Swedish Classification of Healthcare Interventions

**Code**	**Swedish Term**	**English Translation**
AAA20	Inläggande av intrakraniell tryckmätare	Insertion of intracranial pressure monitor
AAA25	Inläggande av epidural tryckmätare	Insertion of epidural pressure monitor
AAA27	Inläggande av intracerebral tryckmätare	Insertion of intracerebral pressure monitor
AAA99	Annan diagnostisk intrakraniell operation	Other diagnostic intracranial surgery
AAB30	Utrymning av spontant intrakraniellt hematom	Evacuation of spontaneous intracranial hematoma
AAC00	Stjälkligatur av intrakraniellt aneurysm	Ligation of intracranial aneurysm stalk
AAC05	Proximal ligatur av intrakraniellt aneurysm	Proximal ligation of intracranial aneurysm
AAC10	Väggförstärkning av intrakraniellt aneurysm	Wall reinforcement of intracranial aneurysm
AAC15	Trapping av intrakraniellt aneurysm	Trapping of intracranial aneurysm
AAC20	Intrakraniell kärlanastomos	Intracranial vascular anastomosis
AAC40	Exstirpation av intrakraniell arteriovenös missbildning	Excision of intracranial arteriovenous malformation
AAC99	Annan intrakraniell kärloperation	Other intracranial vascular surgery
AAD00	Utrymning av epiduralt hematom	Evacuation of epidural hematoma
AAD05	Utrymning av akut subduralt hematom	Evacuation of acute subdural hematoma
AAD10	Utrymning av kroniskt subduralt hematom	Evacuation of chronic subdural hematoma
AAD15	Utrymning av traumatiskt intracerebralt hematom	Evacuation of traumatic intracerebral hematoma
AAD99	Annan operation p.g.a. kraniell eller intrakraniell traumatisk förändring	Other surgery for cranial or intracranial traumatic injury
AAK80	Partiell ektomi av skalltaket För lindring av akut cerebralt ödem	Partial excision of the skull for relief of acute cerebral edema
AAK99	Annan operation på kranium eller dura	Other surgery on cranium or dura
AAW99	Annan operation på kraniet eller intrakraniell struktur	Other surgery on the cranium or intracranial structure
AAA00	Explorativ kraniotomi	Exploratory craniotomy
AAA50	Intrakraniell endoskopi	Intracranial endoscopy
AAD30	Revision av penetrerande eller perforerande skallskada	Revision of penetrating or perforating skull injury
AAD40	Revision av skallfraktur	Revision of skull fracture
AAE99	Annan kraniobasal operation	Other cranio-basal surgery
AAF00	Ventrikulostomi	Ventriculostomy
AAF05	Ventrikuloperitoneal shunt	Ventriculoperitoneal shunt
AAF10	Lumboperitoneal shunt	Lumboperitoneal shunt
AAF15	Ventrikuloatrial shunt	Ventriculoatrial shunt
AAF40	Anläggande av shunt mellan intrakraniell cysta och peritoneum	Creation of shunt between intracranial cyst and peritoneum
AAF99	Annan shuntoperation på hjärnventrikel eller intrakraniell cysta	Other shunt surgery on brain ventricle or intracranial cyst
AAK00	Kranioplastik	Cranioplasty
AAK10	Duraplastik	Duraplasty

## Abbreviations

**CI**: Confidence Interval
**CT**: Computed Tomography
**d-ICH**: Delayed Intracranial Hemorrhage
**DOAC**: Direct Oral Anticoagulant
**ED**: Emergency Department
**ICH**: Intracranial Hemorrhage
**INR**: Prothrombin time, international normalized ratio
**QR**: Quartile Range
**NICE**: National Institute for Health and Care Excellence
**OAC**: Oral Anticoagulants
**SDH**: Subdural Hematoma
**SNC**: Scandinavian Neurotrauma Committee
